# Repression of RNA Polymerase II Transcription by a *Drosophila* Oligopeptide

**DOI:** 10.1371/journal.pone.0002506

**Published:** 2008-06-25

**Authors:** Gyula Timinszky, Miriam Bortfeld, Andreas G. Ladurner

**Affiliations:** 1 Gene Expression Unit, European Molecular Biology Laboratory, Heidelberg, Germany; 2 Structural and Computational Biology Unit, European Molecular Biology Laboratory, Heidelberg, Germany; Cairo University, Egypt

## Abstract

**Background:**

Germline progenitors resist signals that promote differentiation into somatic cells. This occurs through the transient repression in primordial germ cells of RNA polymerase II, specifically by disrupting Ser2 phosphorylation on its C-terminal domain.

**Methodology/Principal Findings:**

Here we show that contrary to expectation the *Drosophila* polar granule component (*pgc*) gene functions as a protein rather than a non-coding RNA. Surprisingly, *pgc* encodes a 71-residue, dimeric, alpha-helical oligopeptide repressor. *In vivo* data show that Pgc ablates Ser2 phosphorylation of the RNA polymerase II C-terminal domain and completely suppresses early zygotic transcription in the soma.

**Conclusions/Significance:**

We thus identify *pgc* as a novel oligopeptide that readily inhibits gene expression. Germ cell repression of transcription in *Drosophila* is thus catalyzed by a small inhibitor protein.

## Introduction

Germline progenitor cells resist signals that promote differentiation into somatic cells during development, thus maintaining their cell fate. In the fruit fly *Drosophila* and in many other species, this occurs through the transient repression of RNA polymerase II (Pol II) mediated transcription in primordial germ cells [1]; [2]. Specifically, this occurs by disrupting the phosphorylation of Ser2 in the C-terminal domain (CTD) of Pol II, a post-translational modification that promotes transcriptional elongation of the polymerase. A screen in *Drosophila melanogaster* identified *polar granule component* (*pgc*) as the gene required for Pol II inhibition and germ cell establishment [3]. *pgc* encodes two small ORFs that diverge within the *D. sophophora* subgenus and have no obvious orthologues outside of the genus. These facts, as well as an ORF1 start codon in an unfavourable translation initiation context and poor codon usage for ORF2, led to the hypothesis that *pgc* functions as a transcription-repressing non-coding RNA [1]; [3].

**Figure 1:**
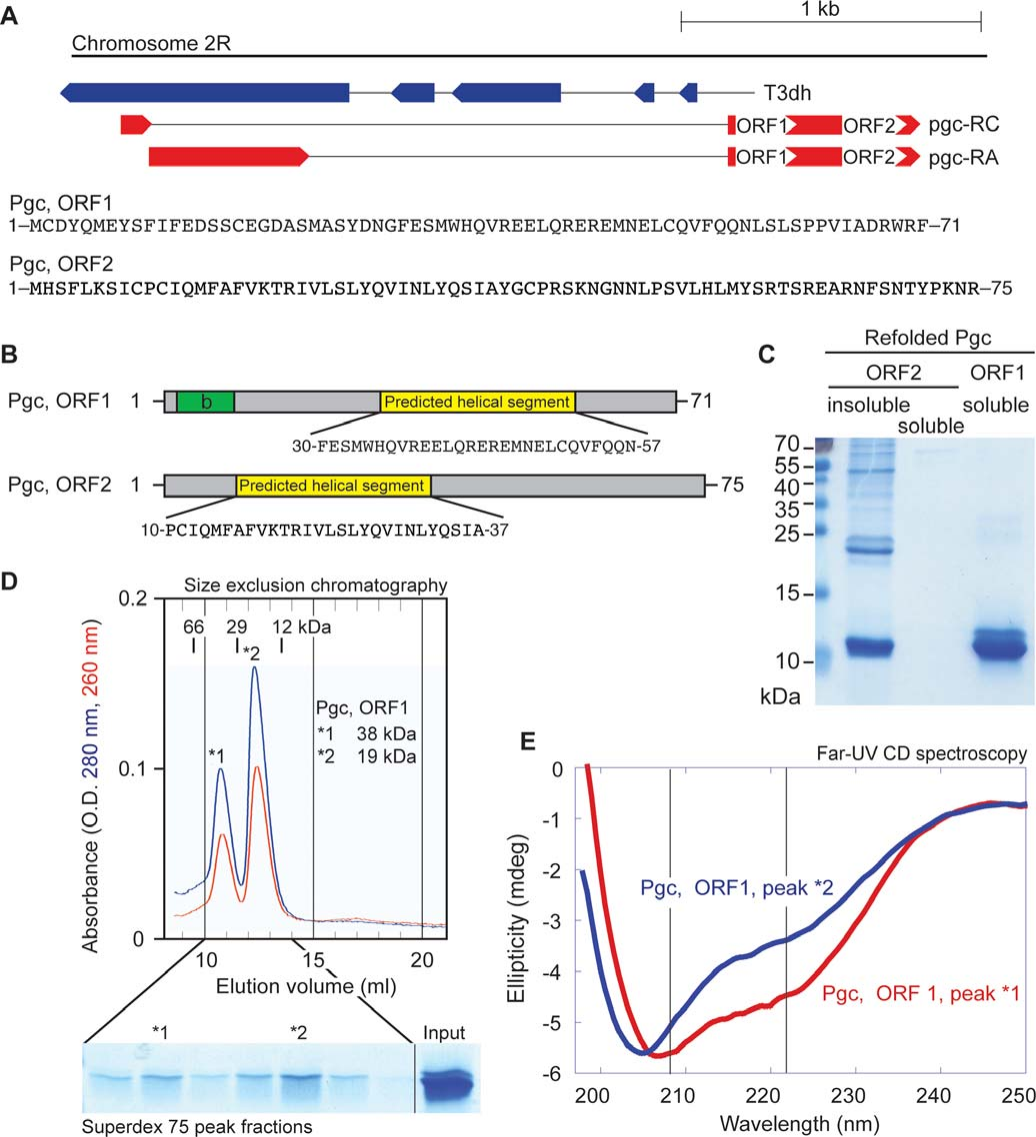
The *pgc* locus encodes an alpha-helical monomeric and dimeric oligopeptide protein. (A) *pgc* contains two open reading frames, encoding potential 71 and 75 residue oligopeptide proteins (Pgc ORF1 and ORF2, respectively). (B) The two candidate oligopeptides are predicted to contain alpha-helical secondary structure. (C) *E. coli* expressed and purified Pgc ORF1 is a soluble protein, in contrast to ORF2, which fails to refold *in vitro*. (D) Size-exclusion chromatography of PGC ORF1 reveals two chromatographic species, one migrating at 18 kDa, the other at 36 kDa, consistent with monomer and dimer fractions of (His)_6_-tagged Pgc ORF1. Absorbance signal at 280 nm (blue) and 260 nm (red). (E) Far-UV circular dichroism spectroscopy spectrum of purified, recombinant PGC ORF1 reveals that recombinant Pgc ORF1 protein contains intrinsic alpha-helical structure. Peak *1 (red), corresponding to the dimeric Pgc ORF1 complex, contains a higher alpha-helical content than monomeric Pgc1 (peak *2, blue).

In a bioinformatic search for novel, small proteins that may be involved as cofactors in transcription-related processes, we noted that *pgc* ORF1 and ORF2 could encode two 71-residue and 75-residue oligopeptides (Figure 1A) with significant predicted alpha-helical structure (Figure 1B). We therefore tested whether these two ORFs may encode folded proteins and directly assessed whether Pgc protein may be responsible for the known repressive roles of the *Drosophila pgc* gene. Transfection assays clearly reveal the ability of *pgc* ORF1 to repress transcription. Further, microinjection of a folded 71 amino acid ORF1 oligopeptide readily inhibits zygotic transcription, establishing Pgc protein as a general transcriptional repressor protein.

## Results and Discussion

### *pgc* ORF1 encodes a small, alpha-helical protein

To test whether *pgc* ORFs encode folded proteins, we recombinantly expressed and purified the two short ORFs encoded by *Drosophila pgc* gene in *E. coli* as (His)_6_-tagged fusion proteins. While ORF2 is insoluble, Pgc ORF1 can be refolded and migrates as an estimated monomer and dimer fraction on a size-exclusion chromatography column (Figure 1C,D). Furthermore, far-UV CD assays reveal alpha-helical structure in both monomer and dimer Pgc fractions (Figure 1E). Dimeric Pgc ORF1 contains a higher helical content, as seen by the lower ellipticity at 222 nm wavelength (dimeric Pgc exhibits a far-UV CD spectrum consistent with ∼20% α-helix and ∼25% β-strand content). Thus Pgc may exist in a dynamic equilibrium between monomer-dimer species. Consistent with what is often seen for very small proteins, our data suggest that Pgc ORF1 dimerization may stabilize the protein's fold.

### The Pgc oligopeptide represses Pol II Ser2 phosphorylation

**Figure 2:**
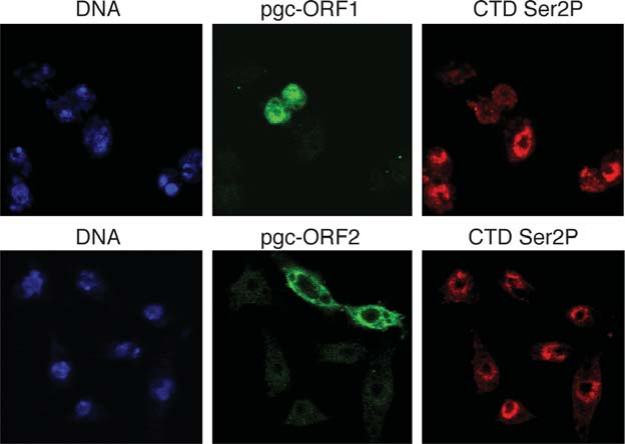
Pgc ORF1 abolishes Ser2 phosphorylation on the RNA polymerase II CTD. Transfected *Drosophila* Kc cells fixed one day after inducing the expression of V5-(His)_6_-tagged Pgc ORF1 and ORF2. Pgc-expressing cells are revealed by anti-V5 antibody (middle panels). Upper panels show that Pgc ORF1 decreases CTD Ser2 phosphorylation (detected using H5 monoclonal anti-phospho-Ser2 antibody). There is no change in CTD Ser2 phosphorylation upon expression of Pgc ORF2 (lower panels).

To test whether a short DNA construct encoding Pgc ORF1 (but not the remainder of the *pgc* sequence) is able to reduce nuclear Ser2 CTD phosphorylation, we transfected *Drosophila* Kc cells with V5-(His)_6_-tagged *pgc* ORF1 and ORF2. *pgc* ORF1 represses Ser2 CTD phosphorylation (Figure 2), while *pgc* ORF2 does not change the Ser2 phosphorylation state of Pol II. The loss of CTD Ser2 phosphorylation suggests Pgc ORF1 directly or indirectly functions by repressing normal Pol II function.

**Figure 3:**
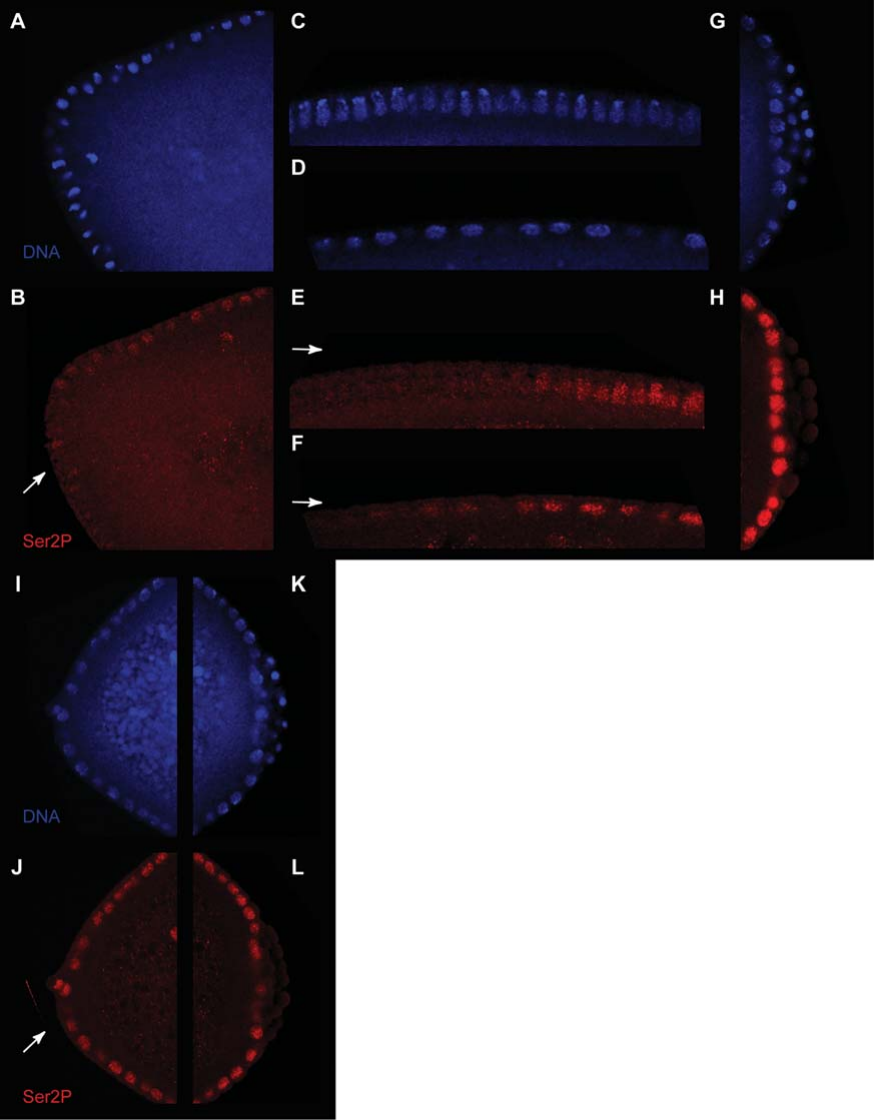
Microinjection of recombinant Pgc protein strongly reduces CTD Ser2 phosphorylation in *Drosophila* embryonic nuclei. Pgc was injected (arrows) into the anterior region of stage 3–4 *Drosophila* embryos. Representative images of embryos fixed 30 minutes after the injection of Pgc protein (A–H) or buffer only (I–L). DNA detected by Hoechst stain (purple, A, C, D, G, I, K) and CTD phospho-Ser2 (green, B, E, F, H, J, L). Ser2 phosphorylation strongly decreases at the site of injection (arrow, B). In comparison, somatic cell nuclei in the posterior area of the same embryo show no changes in Ser2 phosphorylation (H). Ser2 phosphorylation is gradually lost when moving away from the site of injection (E, F, arrow, left, denotes anterior region). Ser2 phosphorylation does not decrease with control injections (J) when compared to the uninjected area (L).

In order to directly test whether Pgc protein, rather than its RNA message, is responsible for the decrease in CTD Ser2 phosphorylation *in vivo*, we micro-injected the anterior pole of stage 3–4 *Drosophila* embryos with folded Pgc. Remarkably, recombinant Pgc suppresses Ser2 CTD phosphorylation around the injection site (Figure 3A–H), but does not alter normal levels of CTD phosphorylation at the posterior pole. Hence, Pgc protein can lower Ser2 CTD phosphorylation levels in somatic embryonic nuclei.

### Pgc protein silences zygotic transcription in the soma

**Figure 4:**
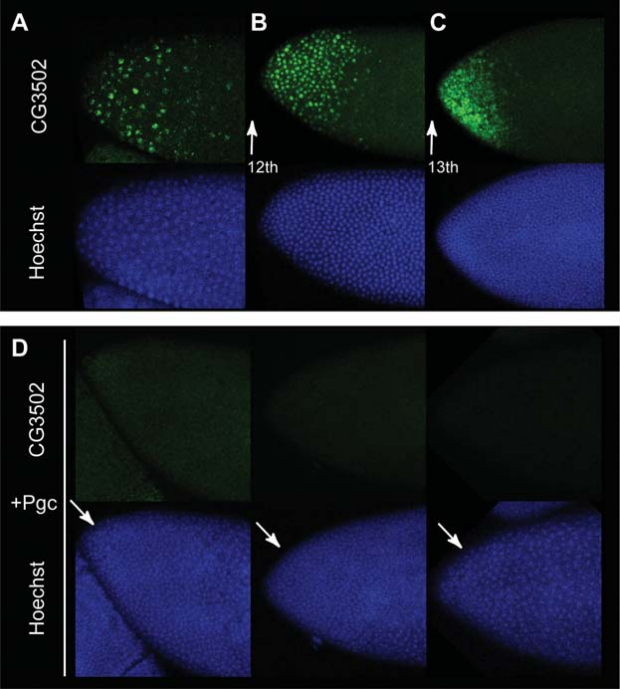
The 71-residue Pgc oligopeptide readily suppresses an early zygotic transcript in *Drosophila* somatic nuclei. (A) Fluorescent *in situ* hybridization with an antisense RNA probe for *CG3502*, a transcript in the anterior region of the embryo whose expression starts during the 11^th^ cleavage cycle. *CG3502* mRNA accumulates in the nucleus before the 13^th^ cleavage division (A, B) and redistributes to the cytoplasm during cellularization (C). (D) No detectable *CG3502* mRNA one hour after purified Pgc is injected into the anterior region of the embryo. The three representative embryos are developmentally beyond the 11^th^ cleavage division and should thus be expressing *CG3502*.

**Figure 5:**
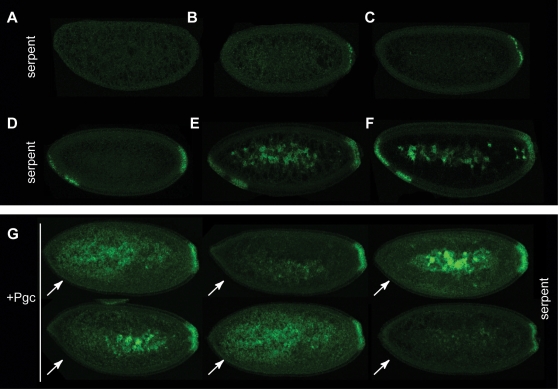
Suppression of a later zygotic transcript in *Drosophila* somatic nuclei by the Pgc oligopeptide. Fluorescent *in situ* hybridization with an RNA probe detecting *serpent* mRNA. There is no detectable maternal supply of *serpent* mRNA (A). *serpent* mRNA is first detectable at the posterior end during cleavage cycle 11 (B). The amount of *serpent* mRNA increases further until cellularization in the posterior somatic nuclei without detectable expression in the anterior nuclei (C). Upon cellularization, *serpent* mRNA is detected in two anterior areas: in cells in the anterior pole and in an antero-ventral cell group (D). As cellularization progresses, the cells become columnar, and *serpent* mRNA signals further increase in the anterior cell groups (E, F). (G) Representative images of cellularizing embryos that have been injected with purified Pgc in the anterior region (arrows). Pgc injection suppresses *serpent* mRNA accumulation in the anterior region (compare with D–F).

To test whether this inhibitory effect on Pol II phosphorylation also alters the transcription of early zygotic genes, as would be predicted for such a dramatic change in CTD Ser2 phosphorylation, we probed Pgc-injected embryos for the presence of two early transcripts [4]. *CG3502*, whose expression starts after the 11th cleavage division before cellularization, and *serpent*, whose expression in the anterior pole starts at cellularization following the 13th cleavage division. Injection of recombinant Pgc into the anterior pole fully represses the expression of *CG3502* (Figure 4). Similarly, *serpent* mRNA is not detected near the injection site, while posterior pole *serpent* RNA accumulates normally (Figure 5). These two assays show that Pgc protein can account for the known biological roles of the *Drosophila pgc* gene. In summary, our data identify a novel, short *Drosophila* oligopeptide protein that can efficiently repress zygotic Pol II transcription in the somatic nuclei of the early embryo. This suggests that *pgc* encodes a folded oligopeptide which renders primordial germ cells insensitive toward somatic differentiation signals by lowering Ser2 CTD phosphorylation and hence blocking Pol II-mediated transcription.

A recent publication by Hanyu-Nakamura and colleagues complements our oligopeptide-focused analysis through of a range of genetic experiments [5]. Further, their studies provide a molecular hint on the direct or indirect target of Pgc's repressive action. Specifically, they identify the Ser2 CTD kinase Cdk9, a subunit of the positive elongation factor P-TEFb, as the likely biological target of Pgc function [5]. Pgc fractionates with Cdk9 in immunoprecipitation assays and affects Cdk9 recruitment to polytene chromosomes, but does not appear to directly inhibit P-TEFb CTD kinase activity *in vitro* [5]. This suggests that Pgc may sequester Cdk9 activity away from active promoters. It is currently not known whether Pgc can interact with Cdk9 directly. Future studies will address the exact mechanism of how Pgc leads to transcriptional inhibition through repression of Pol II CTD Ser2 phosphorylation. It will be interesting, for example, to test whether Pgc alters the ability of the Pol II CTD to interact with transcriptional elongation and other factors, such as the RNA processing machinery. High-resolution structural analysis of Pgc monomers and dimers, for example, should provide important clues about the protein's molecular form and function, and improve the chances of identifying a conserved molecular structure capable of inhibiting Pol II in species beyond *Drosophila*.

Our results show that Pgc is an independently-folded oligopeptide protein which reduces gene expression by affecting Pol II CTD phosphorylation. There are other examples of small, oligopeptide proteins and peptide motifs that regulate gene expression, notably in the RNAi pathway [6]–[8]. Our study identifies a small, 71-residue germ-cell oligopeptide that can critically regulate gene expression at one of the earliest steps on the pathway from gene to protein by suppressing the activity of a co-factor that facilitates transcription. The ability of folded Pgc to inhibit zygotic transcription upon microinjection suggests it is able to dynamically associate with (or sequester) cellular, nuclear target proteins, including the multisubunit transcriptional co-activator P-TEFb, and lead to a rapid and likely reversible repression of RNA Pol II Ser2 CTD phosphorylation.

## Materials and Methods

### Secondary structure predictions

ORF1 and ORF2 of *Drosophila pgc* were analyzed using a combination of secondary structure prediction programmes, including PredictProtein (www.predictprotein.org), IUPred (*iupred.enzim.hu*), GlobPlot 2 (*globplot.embl.de*) and Agadir [9].

### Cloning

*pgc* ORF1 and ORF2 were PCR-amplified from BDGP clone RE14873 using Phusion DNA polymerase (Finnzymes). For bacterial expression of N-terminal (His)_6_-tagged proteins, Pgc was cloned into pETM-11 (a pET-24 derivative) using NcoI and NotI. For inducible expression in *Drosophila* cell culture, ORF1 and ORF2 were cloned into pMT-V5-His (Invitrogen) using EcoRI and NotI. The resulting Pgc proteins are V5 and His-tagged on the C-terminus. Constructs were verified by DNA sequencing.

### *Drosophila* cell culture and transfections

*Drosophila Kc* cells were grown at 25°C in Schneider's *Drosophila* Medium (Invitrogen) supplemented with 10% heat-inactivated fetal bovine serum (Invitrogen), as well as penicillin and streptomycin. For the transfection of *Kc* cells with the pMT-Pgc-V5His constructs, we used Effectene (Qiagen) according to the manufacturer's instructions. We induced Pgc expression with 70 µM CuSO_4_ (Sigma) 16 hours after transfection and fixed the cells 24 hours after induction.

### Pgc protein expression and purification

His-Pgc protein was expressed in *E. coli* Rosetta (Novagen) cells at 37°C for 4 hours and purified from inclusion bodies on a Ni^2+^-NTA column (GE Life Sciences) under denaturing conditions. The bacterial pellet was resuspended and lysed in a buffer containing 50 mM Tris pH 8.0, 10 mM MgCl_2_, 1 mM DTT, 1 mM PMSF and protease inhibitors. The resuspended cells were centrifuged in a SS34 rotor at 9100 rpm at 4°C for 1 hour. The pellet was resuspended in a buffer containing 6 M guanidine-HCl, 50 mM Tris pH 8.0, 250 mM NaCl and extracted for 12–14 hours at 4°C. The suspension was centrifuged in a SS34 rotor at 18000 rpm at 4°C for 2 hours and the supernatant was filtered through 5 µm and 0.22 µm filters. The extract was diluted 1∶10 in equilibration buffer (6 M Urea, 50 mM Tris pH 8.0, 250 mM NaCl, 15 mM Imidazole, 5 mM β-mercaptoethanol) and loaded onto a HisTrap HP (GE Healthcare) column. After loading, the resin was washed with 8 column volumes of equilibration buffer. The bound protein was eluted with equilibration buffer containing 500 mM Imidazole. The eluate was diluted to 1 mg/ml in equilibration buffer and dialyzed in 1 L of a buffer containing 5 M Urea, 5% glycerol, 250 mM NaCl, 50 mM Na_2_HPO_4_/NaH_2_PO_4_ pH 8.0, 5 mM DTT and 25 mM Tris pH 8.0 at 4°C using Spectra/Por (Spectrum) dialysis membranes with a cut off of 3.5 kDa. After 1 hour of dialysis, the protein was dialyzed overnight into PBS containing 5% glycerol. Recombinant Pgc is >99% nucleic-acid free, as determined by absorbance measurements at 260/280 nm wavelength. Pgc protein was stored at 4°C. The biological activity of Pgc was measured by microinjecting embryos with refolded Pgc and determining the inhibition of Ser2 phosphorylation in the CTD of Pol II.

### *Drosophila* embryo injections

For the injection of *Drosophila* embryos with bacterially purified Pgc (200 µM) in PBS containing 5% glycerol, 0–1 hour old embryos were collected from *white^1118^* flies kept on apple juice agar at 19°C and 65–70% humidity. Each embryo was injected with 100–200 picolitre of Pgc. For control experiments, we injected the same volume of dialysis buffer (PBS). The eggs were aged at 19°C and 65–70% humidity until fixation and prepared for injection using standard protocols. Before injection, they were dechorionated in 50% bleach, desiccated and covered with 10S Voltalef oil (Atochem). For injections, we used Femtotips I microinjection needles (Eppendorf) on an Eppendorf microinjector. Injected embryos were aged further before fixation. To analyze the effect of refolded Pgc on CTD Ser 2 phosphorylation, we injected 2–3 hour old embryos and aged them 30 minutes after injection until cellularization was observed in the majority of the injected embryos. For RNA *in-situ* hybridizations, embryos were aged 1 hour following Pgc injection. Pre-cellularization embryos were staged by counting Hoechst-stained nuclei, while later stages were determined by measuring the average length of elongating nuclei (from 3–4 to 10–15 µm).

### Immunostainings

We used standard protocols to perform immunostainings [10]. *Kc* cells were fixed in 4% formaldehyde for 10 minutes, washed in PBS-Tween 20 (0.05%) and blocked in 5% milk in PBS-Tween 20 for 1 hour. *Drosophila* embryos were fixed by rotating them in 1 part heptane (Sigma)∶1 part 4% formaldehyde for 10 minutes and devitellinized in 1 part heptane∶1 part methanol (Sigma) with vigorous shaking for 1 minute. Devitellinized embryos were washed in methanol, rehydrated in PBS-Tween 20 (0.5%) and blocked in 5% milk in PBS-Tween 20 for 1 hour. We incubated the fixed cells and embryos with the primary antibodies at 4°C overnight and the secondary antibodies at room temperature for 3 hours. We used mouse monoclonal anti-phospho-Serine2 CTD (H5) antibody (Covance; at 1∶800 dilution) and rabbit polyclonal anti-V5 antibody (Abcam at 1∶1000 dilution). Hoechst stain labelled DNA. The samples were mounted using VectaShield mounting medium.

### RNA *in situ* hybridizations of somatic transcripts

Fluorescent *in situ* hybridizations was performed as described by the Krause laboratory (http://www.utoronto.ca/krause/). DIG-labeled RNA probes were made through PCR amplification of BDGP DGC clones LD34564 and LD16058 using T7, T3 or SP6 primers (Invitrogen) and subsequent *in vitro* transcription using T7, T3 or SP6 RNA polymerases (Fermentas) and DIG RNA-labelling mix (Roche). We used biotin-conjugated mouse monoclonal anti-DIG antibody (Jackson ImmunoResearch) and Alexa Fluor 488 TSA Kit T-20932 (Invitrogen) for signal amplification. All imaging with a Leica TCS SP2 AOBS (Leica Microsystems) confocal microscope.
